# Leiomyoma of the tunica albuginea, a case report of a rare tumour of the testis and review of the literature

**DOI:** 10.1186/1746-1596-7-140

**Published:** 2012-10-09

**Authors:** Felix Bremmer, Felix J Kessel, Carl L Behnes, Lutz Trojan, Elmar Heinrich

**Affiliations:** 1 Department of Pathology, University Medical Centre Göttingen, Robert-Koch-Str. 40, Göttingen, 37075, Germany; 2 Department of Urology, University Medical Center Göttingen, Robert-Koch-Str. 40, Göttingen, 37075, Germany

**Keywords:** Leiomyoma, Tunica albuginea, Immunohistochemistry

## Abstract

**Background:**

Leiomyomas are benign tumours that originate from smooth muscles. They are often seen in the uterus, but also in the renal pelvis, bladder, spermatic cord, epididymis, prostate, scrotum or the glans penis. Leiomyomas of the tunica albuginea are extremely rare.

**Case presentation:**

A 59-year-old white male has noted an asymptomatic tumour on the right side of his scrotal sac for several years. This tumour has increased slowly and caused local scrotal pain. An inguinal incision was performed, in which the hypoplastic testis, the epididymis and the tumour could be easily mobilized. Macroscopically the tumour showed a solid round nonencapsulated whorling cut surface. Histologically the diagnosis of a leiomyoma was made.

**Conclusion:**

We report here a very interesting and rare case of a leiomyoma of the tunica albuginea. Leiomyomas can be a possible differential diagnosis in this area.

**Virtual Slides:**

http://www.diagnosticpathology.diagnomx.eu/vs/2585095378537599

## Background

Leiomyomas are benign tumours that originate from smooth muscles cells and are often found as benign lesions arising in the uterus [1,2]. But there are also been seen cases of leiomyomas of the renal pelvis, bladder, spermatic cord, epididymis, prostate, scrotum and the glans penis [1,3-6]. Rare cases of a primary ovarian leiomyoma [7] , leiomyoma of the testis [8] or leiomyoma of the kidney have been also reported [9]. Leiomyomas of the tunica albuginea are extremely rare, and to our knowledge only five cases have been reported so far [10-15]. In case of a bilateral leiomyoma so far only two cases are reported [14]. Here we present a case of a leiomyoma of the tunica albuginea.

### Case History

#### Clinical features

A 59-year-old white male has noted an asymptomatic tumour on the right side of his scrotal sac for several years. Since the size of this tumour has increased and lately sometimes even caused local scrotal pain, he was presented to the clinic of urology.

Physical examination revealed a solid tumour, approximately 5 cm in diameter, on the right scrotal side. The testis on this side felt unremarkable, though it seemed to be very small. Inguinal lymph nodes were not palpable.

**Figure 1:**
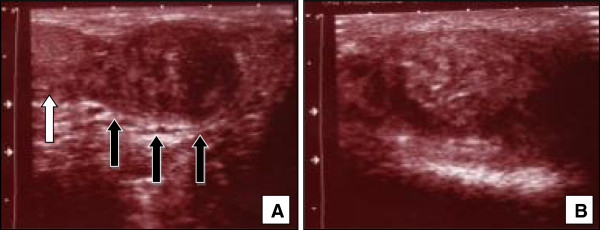
**In sonographic examination beside a little hypoplastic but unremarkable testis (white arrow) a 5 cm in diamater tumour could be seen (black arrows) (A).** The tumour shows echogenic and cystic areas **(B).**

Ultrasound of the scrotum revealed a tumour with both echogenic and cystic areas and a diameter of 4cm on the right scrotal side. The testis was hypoplastic but unsuspicious (Figure 1). Whether testis or epididymis were affected by the tumour could not be clearly seen in ultrasonography. On the left scrotal side a tumour much smaller in size with a similar sonographic appearance was detected.

Choosing an inguinal incision, the hypoplastic testis, the epididymis and the tumour could be easily mobilized. The resection of the tumour was accomplished without harming testis and epididymis. Testis and epididymis were replaced into the scrotum. The postoperative course was uneventful.

#### Macroscopy and Microscopy

**Figure 2:**
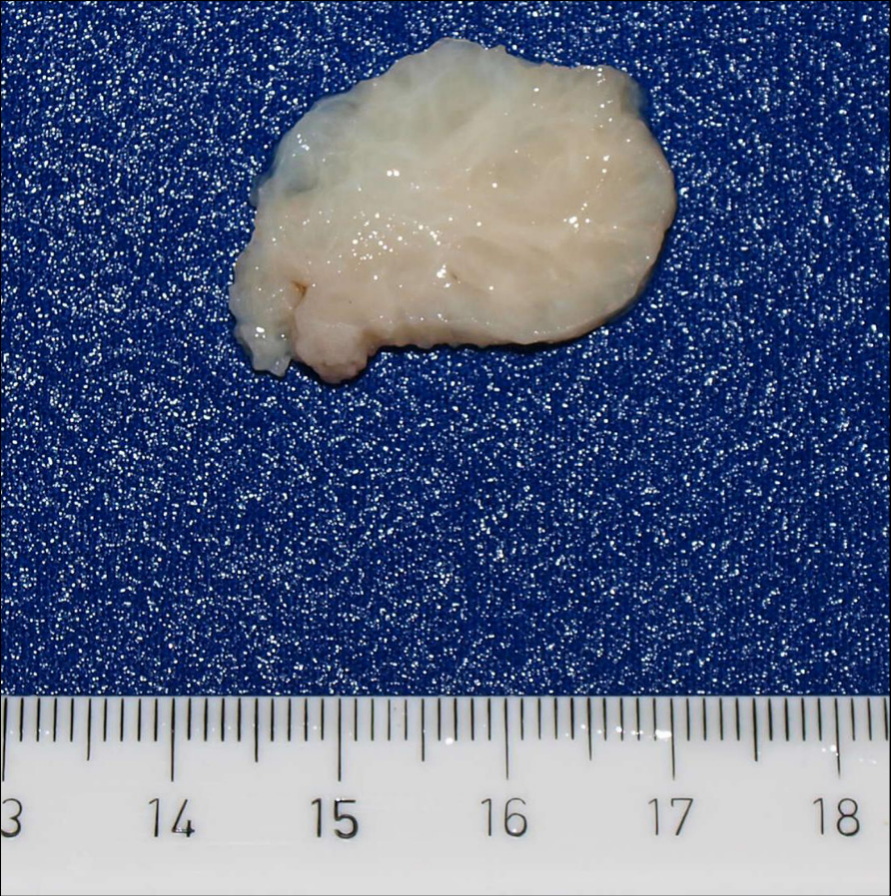
**Appearance of cut surface of the right scrotum.** A nodular tumour with a whorling surface can be seen.

**Figure 3:**
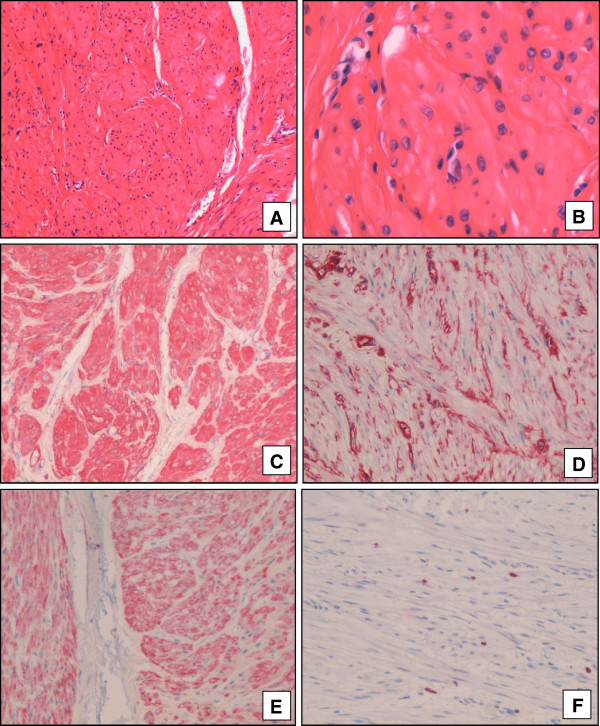
**Histollogically interlacking smooth muscle bundles are seen (A, x40; B, x200).** Immunohistologically Actin **(C, x100)**, Vimentin **(D, x100)** and Desmin **(E, x100)** show positive expression pattern. In the Ki-67 staining no increased proliferation activity could be detected **(F, x100).**

After excision, the tumour tissue was sent to the department of pathology for histological examination. In macroscopical examinantion a solid round nonencapsulated whorling tumour of white colour and a mass of 5 x 3,5x 3.5 cm was seen (Figure 2). The testis and epididymis were not involved. Microscopically, the tumour is composed of interlacing and whorling bundles of smooth muscle cells. In these smooth muscle cells vascular channels are seen (Figure 3 A+B). The tumour cells are spindeled containing a centrally located nucleolus and showing no mitotic activity or nuclear atypia. In immunohsitochemical staining the tumour cells were positive for vimentin, desmin, actin. Keratin and s-100 were negative (Figure 3 C-F).

## Discussion

Leiomyomas are benign tumours orginate from smooth muscles cells. Three different types respective to their origin are known; (1) derivation of arrector pili muscle (piloleiomyoma), (2) derevation of smooth muscles of blood vessels (angioleiomyoma), and (3) genital leiomyoma (p.e. from the smooth muscles of the scrotum) [16]. Tumours of mesenchymal origin in the scrotum are rare, more often cutaneous epithelial tumours are seen [13]. Patients with a leiomyoma of the tunica albuginea are usually about the sixth decades [10-15]. Leiomyomas of the tunica albuginea considered to be of benign behaviour. It shows no invasive growth or metastasis [14]. In sonographic investigations potential differential diagnosis are inflammatory hydrocele, and multiloculated hematocele [17] and a Sertoli cell tumour of the testis [18]. Tumours arising from the testicular tunics are rare and in the most cases fibromas [12]. The potentially aetiology of leiomyomas of the tunica albuginea is discussed controversial. They could arise from the smooth muscle from blood vessels [12] but also may be from totipotent teratoma [10]. Possible differential diagnosis is an inflammatory myofibroblastic tumor (IMT) of the spermatic cord [19]. IMTs are also seen in the lung [20] and are neoplasms of proliferating myofibroblasts, with a variable inflammatory component [19]. Immunohistochemically leiomyomas of the tunica albuginea are positive for Desmin as we could show in the present case report, IMTs are negative [19]. Thus, Desmin is useful to distinguish these two tumours. An orchiectomy is not necessary and instantaneous section should be performed while operation. In this case, the tumour is growing slowly and compressed surrounding structures, causing a hypoplastic testis. Interestingly in ultrasound a second lesion on the other testis from the same appearance could be seen. The patient disagreed excising this lesion because of absent pain in this area. Probably this case is also a bilateral leiomyoma. As the patient did not agree for surgery on the left side, we probably will report in a few years in case of tumour progress and consecutive pain, which was the reason for the patient for surgery on the right side.

## Conclusions

This is an extremely rare and interesting case of a leiomyoma of the tunica albuginea. As far as we know only 5 cases of this tumour of the tunica albuginea have been reported. Leiomyomas in this area increasing slowly and may cause scrotal pain. Leiomyomas can be a possible differential diagnosis in this area.

### Consent

Written informed consent was obtained from the patient for publication of this case report and any accompanying images. A copy of the written consent is available for review by the Editor-in-Chief of this journal.

## Competing interests

The authors declare that they have no competing interests.

## Authors’ contributions

FB and FJK drafted the manuscript and were responsible for the interpretation of the data. FB and CLB were responsible for microscopic and histopathologic elements. LT, FK and EH cared for the patients. EH was responsible for the critical revision of the manuscript. All authors read and approved the final manuscript.
